# A Method of Bleaching Sponges

**Published:** 1892-11

**Authors:** H. Morell

**Affiliations:** Toronto, Canada


### A Method of Bleaching Sponges.

In reply to "Reader" I herewith give a good method of bleaching sponges. The sponges should be freed from lime by immersing them in dilute hydrochloric acid. They then should be soaked in a 1 per cent. solution of permanganate of potash, removed and washed thoroughly with water; now press them dry; they are now to be put into a solution of one-half pound of hyposulphite of sodium in one gallon of water, to which one ounce of oxalic acid has been added; have them in this solution for fifteen minutes. Finally take them out and wash thoroughly. By this treatment the sponges are rendered perfectly white and remain so. Gerster advises the sponges to be kept in a five per cent. solution of carbolic acid until required for use. Sponges once used should be carefully washed with green soap and hot water before being put away again.

Toronto, Canada. H. Morell, M.D., C.M.

